# Advancing the science of microbial symbiosis to support invasive species management: a case study on *Phragmites* in the Great Lakes

**DOI:** 10.3389/fmicb.2015.00095

**Published:** 2015-02-19

**Authors:** Kurt P. Kowalski, Charles Bacon, Wesley Bickford, Heather Braun, Keith Clay, Michèle Leduc-Lapierre, Elizabeth Lillard, Melissa K. McCormick, Eric Nelson, Monica Torres, James White, Douglas A. Wilcox

**Affiliations:** ^1^U.S. Geological Survey, Great Lakes Science CenterAnn Arbor, MI, USA; ^2^U.S. Department of Agriculture, Agricultural Research ServiceAthens, GA, USA; ^3^Great Lakes CommissionAnn Arbor, MI, USA; ^4^Department of Biology, Indiana UniversityBloomington, IN, USA; ^5^Smithsonian Environmental Research CenterEdgewater, MD, USA; ^6^Department of Plant Pathology and Plant-Microbe Biology, Cornell UniversityIthaca, NY, USA; ^7^Department of Plant Biology and Pathology, Rutgers UniversityNew Brunswick, NJ, USA; ^8^Department of Environmental Science and Biology, The College at Brockport, State University of New YorkBrockport, NY, USA

**Keywords:** symbiosis, *Phragmites*, invasive species management, fungi, bacteria, collaborative, endophyte, Great Lakes Region

## Abstract

A growing body of literature supports microbial symbiosis as a foundational principle for the competitive success of invasive plant species. Further exploration of the relationships between invasive species and their associated microbiomes, as well as the interactions with the microbiomes of native species, can lead to key new insights into invasive success and potentially new and effective control approaches. In this manuscript, we review microbial relationships with plants, outline steps necessary to develop invasive species control strategies that are based on those relationships, and use the invasive plant species *Phragmites australis* (common reed) as an example of how development of microbial-based control strategies can be enhanced using a collective impact approach. The proposed science agenda, developed by the Collaborative for Microbial Symbiosis and *Phragmites* Management, contains a foundation of sequential steps and mutually-reinforcing tasks to guide the development of microbial-based control strategies for *Phragmites* and other invasive species. Just as the science of plant-microbial symbiosis can be transferred for use in other invasive species, so too can the model of collective impact be applied to other avenues of research and management.

## Introduction

Invasion of native ecosystems by non-native (i.e., exotic) plant species is a widespread problem. For example, Morse et al. (1995) estimated that more than 5000 exotic plant species have become established and displaced native plant species in the U.S. The problem continues to grow as over 700,000 hectares per year of wildlife habitat are invaded by invasive species (Babbitt, 1998). Invasive plants negatively impact both the ecosystems and the economy of the United States (Pimentel et al., 2000), where about 400 of the 958 species listed as endangered or threatened are considered to be at risk due to pressure from invasive species (Wilcove et al., 1998). Management and control of invasive plants is a priority for many agencies and organizations across the United States and entails a significant investment of resources. For example, the National Invasive Plants Council, composed of members of many federal agencies with a goal to provide high-level interdepartmental coordination of federal invasive species actions, estimated that $2.2 billion (U.S.) was spent during FY2012 on invasive species activities (National Invasive Species Council, 2014). The total control cost for exotic and invasive aquatic weeds in the United States is estimated at $100 million annually (Pimentel, 2005). In the State of Florida alone, $14.5 million is spent annually on aquatic hydrilla (*Hydrilla verticillata*) control, and *H. verticillata* infestations in only two Florida lakes have amounted to $10 million annually in recreational losses, including swimming and boating (Center et al., 1997). Similarly, state departments of natural resources, various collaboratives, and local watershed councils are also concerned with invasive species. In the Great Lakes region, the Great Lakes Restoration Initiative (GLRI), the largest U. S. investment in the Great Lakes in two decades, includes combating invasive species as one of its five urgent issues (Great Lakes Restoration Initiative, 2010, 2014).

Although extensive resources from state and federal agencies have been devoted to both management and control of invasive plant species across the U.S., there is evidence that this intensive investment may not be producing the intended management results (Reid et al., 2009; Martin and Blossey, 2013). There is a need for new, innovative tools to control invasive species that address the drivers of invasion. A growing body of literature supports microbial symbiosis as a foundational principle for the competitive success of invasive species. Much of this insight has emerged from ecological studies of microbiomes (see Glossary for definitions of select terms) demonstrating that the health, productivity, and adaptive capacities of all organisms, whether they be humans (Pflughoeft and Versalovic, 2012), non-human mammalian species (Ley et al., 2008; Muegge et al., 2011), insects (Engel and Moran, 2013), amphibians (Kohl et al., 2013; Kueneman et al., 2014), birds (Kohl, 2012), fish (Wu et al., 2012; Ye et al., 2014), or plants (Bulgarelli et al., 2013; Berg et al., 2014; Rout, 2014) can be linked in various ways to their microbiomes (i.e., microbial communities). This new and growing understanding of the diversity, specificity, and wide-ranging function and impacts of host-associated microbiomes makes it clear that the behavior, dynamics, and interactions of organisms cannot be understood or predicted without a consideration of their associated microbiota (Gilbert et al., 2012). We believe, therefore, that a deeper understanding of the relationships between invasive species and their associated microbiomes, as well as the interactions with the microbiomes of native species, can lead to key new insights into invasive success and potentially new and effective control approaches. This approach is particularly promising for invasive plant species because of opportunities to target control efforts on the special dependence that all plants have on the recruitment of microbiota for growth, tolerance to stress, and resistance to disease. Potential control efforts could target the introduction of pathogenic microbes or inhibition of beneficial fungi (e.g., targeting microbial relationships that confer competitive benefits). In this manuscript, we review microbial (primarily endophytic) relationships with plants, outline steps necessary to develop invasive species control strategies that are based on those relationships, and use the invasive plant species *Phragmites australis* as an example of how development of microbial-based control strategies can be enhanced using a collective impact approach.

### Plant-microbial interactions

**Figure 1:**
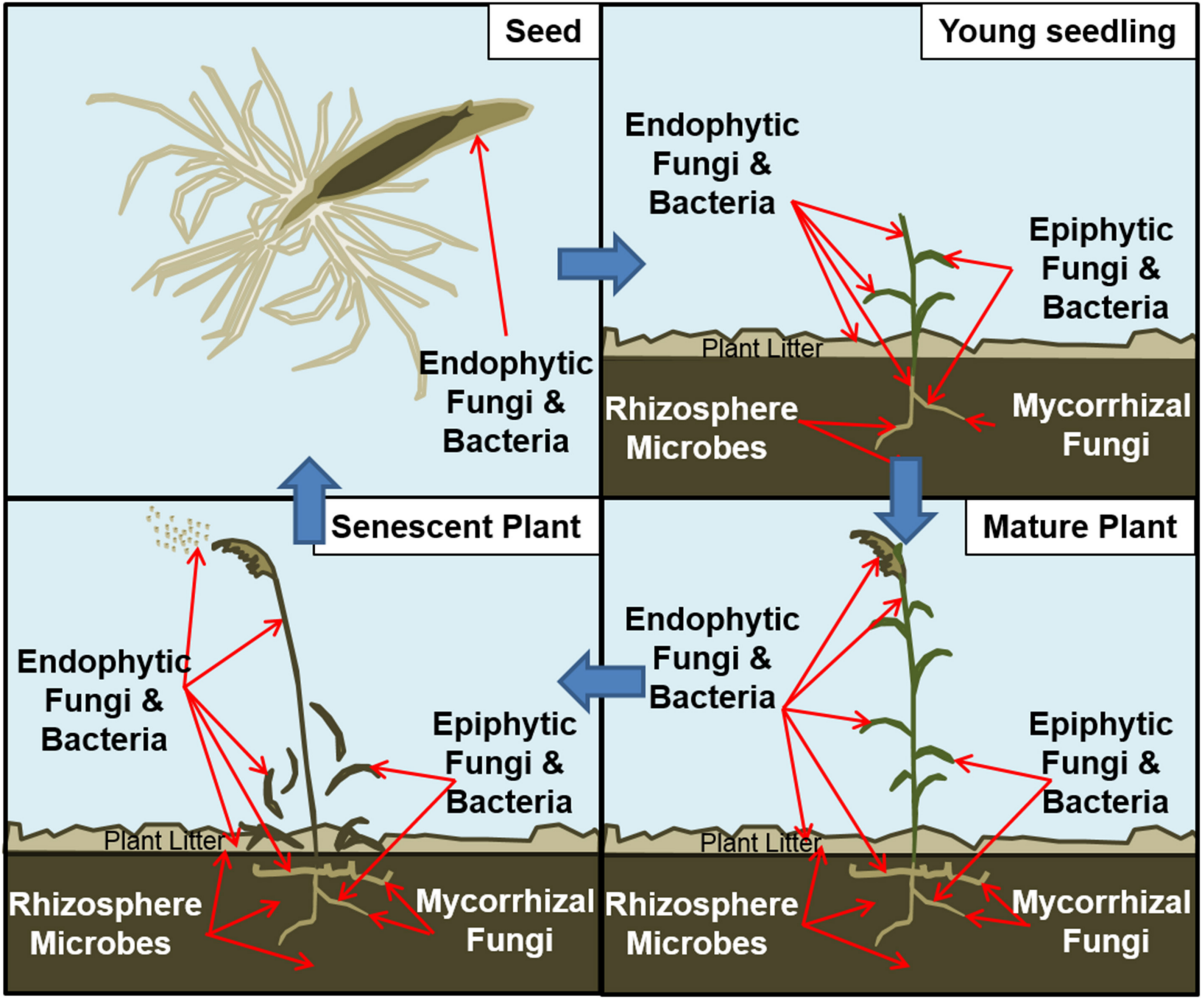
**Schematic of microbiome surrounding a plant throughout its life history**. UL: Endophytic fungi and bacteria can be transmitted within the seed coats of certain plant species. UR: As seeds germinate, roots, stems, and leaves of seedlings can be inhabited by various microbes. Those microbes may have been transmitted through seeds, soil and plant litter on site, or airborne spores. LR: A mature plant may be thoroughly infected with microbes. LL: As perennial plants senesce, some endophytes are transmitted to the next generation through seeds or through living rhizomes. Other microbes may be transmitted through spores in the plant litter.

As with humans and other animals, plants also interact symbiotically with microbes throughout their life history. These symbioses are initiated through vertical transmission to juveniles at the time of seed development or through continuous horizontal acquisition from the environment (Figure 1). This plant-associated microbiome (Turner et al., 2013; Rout, 2014) spans the diversity of microbial life residing either within the plant as endophytes or as epiphytes on foliar (Peñuelas and Terradas, 2014) and subterranean (Mendes et al., 2013) plant surfaces. Broadly, these associations may be either intimate or casual, yet many are thought to contribute to plant health and development (Borer et al., 2013). Many plant-microbe associations may be commensal, for which no overt benefit or harm is observed, or mutualistic, in which plant growth and development are often promoted (Hirsch, 2004). Still other pathogenic symbioses may negatively impact plant growth and/or result in developmental deficiencies or mortality. These relationships also are not static and may vary from mutualistic to pathogenic during different stages of the microbial or plant life cycle (Kogel et al., 2006). Responses of plant populations to this range of symbiotic associations will directly reflect the net impacts of both mutualistic and pathogenic symbioses, as well as indirect impacts that may involve commensals (Bever et al., 2010).

#### Endophytic mutualists and pathogens

The endophytic habit is described as the internal colonization of a plant by a microbe. There are several variations to this endophytic life style. For example, endophytic microbes are often restricted to particular organs, usually roots, stems, or leaves. Some endophytes occupy only above-ground plant parts, whereas others are restricted to subterranean organs and tissues. Endophytic microbes most commonly live exclusively within a plant in a biotrophic mode, obtaining their nutrition solely from the plant. As a result, many endophytic microbes form obligate associations with plants, most often inside the plant host but occasionally outside of the host (e.g., arbuscular mycorrhizal fungi or AMF). Commonly, endophytic microbes that are systemically distributed in plants (Class 1 and 2 fungal endophytes) are vertically transmitted through successive generations of hosts in seeds or in rhizomes of clonal plants, whereas endophytes restricted to particular tissues or organs of hosts (e.g., class 3 endophytes) are generally transmitted horizontally (Rodriguez et al., 2009). Among the more commonly studied fungal endophytes are species of *Epichloë* (=*Neotyphodium* asexual stage) that are restricted to above-ground portions of cool-season grasses, including leaves and inflorescences (Tanaka et al., 2012), and broadly distributed root-infecting AMF that comprise species within the phylum Glomeromycota (Willis et al., 2013), commonly in the genera *Glomus* and *Gigaspora* (Dumbrell et al., 2010). Bacterial endophytes, however, may represent species spanning several bacterial phyla: Actinobacteria, Proteobacteria, Firmicutes, and Bacteroidetes (Malfanova et al., 2013). Endophytic infections by either fungi or bacteria often lead to enhanced plant productivity, either by enhancing nutrient acquisition, producing plant growth hormones, synthesizing metabolites that restrict vertebrate or invertebrate herbivory, or also by reducing disease susceptibility (Rodriguez et al., 2009). However, under appropriate conditions, endophytic interactions may transform from a mutualistic association to a pathogenic association (Newton et al., 2010; Alvarez-Loayza et al., 2011), blurring the lines between species that are strict mutualists and those that are strict pathogens.

Most common epiphytic bacterial and fungal plant pathogens also have a significant endophytic phase to their life cycle. Latent infections are common, and pathogens may reside endophytically in plants for extended periods without causing any mortality, growth reductions, or reductions in fitness (Delaye et al., 2013; Malcolm et al., 2013). Many of these potential pathogens originate from epiphytic populations residing either in the rhizosphere (below-ground zone adjacent to plant roots) or phyllosphere (above-ground zone adjacent to leaves), although pathogen communities ultimately found in roots are more diverse than those found in leaves (Angelini et al., 2012). As with *Epichloë* and other fungal and bacterial endophytes, interactions with plants may switch from pathogenic back to mutualistic, reinforcing the importance of the dynamics of interacting factors associated with hosts, microbes, and the environment (Scholthof, 2007) that ultimately determine the nature of microbial interactions with plants at any given time.

#### Epiphytic microbial associations

The epiphytic lifestyle generally refers to microbial development directly on host surfaces. Yet, this development, driven by carbon release from plant parts (Hirsch et al., 2013), is often maintained within a spatially and temporally variable phyllosphere and the rhizosphere. These epiphytic symbionts originate from soil, water, seed, animal excrement, or the atmosphere and comprise the breadth of bacterial and fungal diversity (Vorholt, 2012). Different plant organs and tissues support different communities of microbes (Normander and Prosser, 2000), but in all cases, these epiphytic associations are driven largely by nutrients released from the plant into the adjacent soil (Dennis et al., 2010) or leached from foliar plant parts (Vorholt, 2012). Similar to endophytic associations, epiphytic associations span the range from mutualistic to pathogenic but often provide positive impacts on plant growth and health either through direct growth enhancements (Lugtenberg and Kamilova, 2009) or suppression of pathogens (Mendes et al., 2011).

### Plant-microbial symbiosis and invasion

Increasingly, it is recognized that microbial symbioses may be important determinants of plant invasiveness and can either exacerbate or inhibit invasive success, depending on origins of the symbiont (from the native or invasive range) and on the direction, prevalence, and strength of the symbiotic interactions (Richardson et al., 2000; Berg et al., 2014; Coats and Rumpho, 2014). However, the nature and magnitude of the role of microbial symbioses in biological invasions is not always clear (van der Putten et al., 2007). Therefore, a better understanding of general mechanisms of biological invasions as a whole will result in more effective management of invasive plant populations (Mack, 1996; Rejmanek, 2000; Richardson et al., 2000).

#### Invasive plants and native pathogens

Darwin (1872) observed that plant and animal species brought to new regions of the world often experienced dramatic population growth and surmised that these species escaped from regulation by “natural enemies.” The enemy release hypothesis (ERH) predicts that plants introduced to a new region will benefit by encountering fewer specialist enemies compared to their native range and will be less affected by resident generalist enemies than resident plants. This escape from natural enemies would provide a competitive advantage over resident species (but see van Kleunen and Fischer, 2009). Many studies suggest that biological invasions are most likely to start in areas with low levels of ecological resistance and by invaders largely free from their native natural enemies (Reinhart et al., 2003; DeWalt et al., 2004; Knevel et al., 2004; Vila et al., 2005) (but see Beckstead and Parker, 2003, for an exception). For example, correlative studies report that many invasive plants are associated with more foliar (Mitchell and Power, 2003) and root (van der Putten et al., 2005) pathogens in their native than non-native ranges. Further, plants categorized as harmful invaders experienced a greater decline in pathogen infection from native to invaded range than weak invaders. Because most natural plant communities have diverse resident pathogens, successful invaders are likely to encounter non-adapted pathogens that cause less damage relative to what they experienced in their native ranges. However, as the density, range, and time-since-invasion of invasive plants increase, interactions with pathogens are likely to change.

#### Invasive plants and novel pathogens

Native pathogens, which may be novel to the introduced species, may immediately prevent invasion (biotic resistance hypothesis) so that the invading species never becomes established or reaches such densities as to displace native species (Elton, 1958; Knevel et al., 2004; Parker and Gilbert, 2004). Outside of agricultural species, we have little knowledge of failed invasions (Scheffer, 1997). Biotic resistance may be more effective where invasive species are closely related to native species. For example, Parker and Gilbert (2004) found no difference in disease levels in native vs. introduced clovers occurring at the same site. Invasive species from an unrelated genus or family should be less likely to be colonized by novel pathogens than invasive species closely related to co-occurring native species. However, over time, the number of novel pathogens that accumulate on invasive species is likely to increase. For example, Strong and Levin (1975) found that introduced British trees support the same number of fungal parasites as native tree species 300 years following their introduction. As the success (i.e., high density) of an invasive species increases, the chance that a virulent pathogen will arise and lead to epidemics and major die-offs also increases. Negative effects of pathogen buildup have been demonstrated both theoretically and empirically (Hudson et al., 1998; Turchin et al., 1999; Hassell, 2000). Disease epidemics in native plant species (Rizzo and Garbelotto, 2003) and rapid control of invasive species by biocontrol efforts (Burdon et al., 1981; Cox and McEvoy, 1991) also point to the potential of enemies to regulate plant populations. For example, the weevil (*Euhrychiopsis lecontei*) colonized Eurasian watermilfoil (*Myriophyllum spicatum*) and greatly reduced populations across its invasive range (Creed and Sheldon, 1995; Creed, 2000). More recently, Flory et al. (2011) reported that a *Bipolaris* fungal pathogen greatly reduced the biomass and reproduction of invasive Japanese stiltgrass (*Microstegium vimineum)* in naturally-infected invasive populations. Over time, invasive plant species may become increasingly regulated by natural enemies (Flory and Clay, 2013).

#### Invasive plants and native mutualists

Most plant species form mutualistic symbioses with arbuscular mycorrhizal fungi (Allen, 1991), N-fixing bacteria (Huss-Danell, 1997; Parker, 2001), or endophytic fungi (Clay and Schardl, 2002; Angelini et al., 2012) or from simultaneous infection by multiple mutualists (Larimer et al., 2010). However, as in the case of the ERH, invasive species may often colonize new habitats without their native symbiont. If a microbial mutualist is obligate, invasions will fail in the absence of the symbiont. For example, early attempts to introduce pines into Australia failed until appropriate mycorrhizal fungi were introduced simultaneously (Allen, 1991). Similarly, Parker (2001) concluded that “legumes may often fail at colonization attempts within habitats where mutualist partners are scarce.” However, this situation can also favor invasive species that are less dependent on mutualistic symbionts. For example, invasive St. John's wort (*Hypericum perforatum)* is less dependent on AMF compared to populations from its native range (Maron et al., 2004). More generally, colonizing species may be less dependent on symbiotic associations than non-colonizing species (Baker and Stebbins, 1965). On the other hand, if colonization by the plant and symbiont occur simultaneously, as in the case of seed-transmitted fungal endophytes of grasses (Clay and Schardl, 2002), then invasiveness may be enhanced by symbiosis. In experimental plots of non-native tall fescue grass (*Lolium arundinaceum*) where endophyte infection was experimentally manipulated, endophyte-infected plots had significantly greater biomass of tall fescue, less biomass of other species, and lower species richness (Clay and Holah, 1999; Rudgers and Clay, 2008). Non seed-transmitted mutualists may be widely dispersed and not limit invasions. For example, in Hawaii the invasive species faya (*Myrica faya)* fixes nitrogen via symbiosis with *Frankia* bacteria and greatly alters ecosystem nitrogen dynamics (Vitousek et al., 1987; Walker and Vitousek, 1991). However, Zimpfer et al. (1999) found that the density of infective *Frankia* decreased with distance from established invasive *Casuarina cunninghamiana* trees in Jamaica, suggesting strong spatial dependence of invasions on *Frankia* density associated with established host populations.

#### Invasive plants and novel mutualists

Invasive plants colonizing habitats in the absence of their native symbionts may become colonized by novel mutualists. The likelihood of this occurring may depend on the level of host-symbiont specificity and on the phylogenetic relationship of the invasive plant with native plant species. Some mutualistic interactions like pollination or seed dispersal may be fairly general and do not represent a strong barrier to invasion (Richardson et al., 2000). One example of an invasive plant that established a symbiotic association with a novel mutualist in its invaded range is purple nutsedge (*Cyperus rotundus*) infected by the fungal symbiont *Balansia cyperi*. The plant is native to Asia but has been widely introduced in agricultural areas outside its native range–to the extent that it is classified as the world's worst weed (Holm et al., 1977). *Balansia cyperi*, on the other hand, is native to the southeastern U.S., Central America, and South America, where it infected several native *Cyperus* species (Diehl, 1950). Invading purple nutsedge populations in the U.S. Gulf coast region were also infected by *B. cyperi*, which produced a large increase in bulbil production and overall plant reproduction (Stovall and Clay, 1988). The fungus likely jumped from a native *Cyperus* host to *C. rotundus* in this region, exacerbating its competitive ability and invasiveness. Host shifts of novel mutualists onto invasive plants must certainly occur in other systems but have not been well-documented.

## Invasive species management through microbiome manipulation

Given the multitude of means by which microbes can impact host organisms and, ultimately invasion success, there is great potential for the management of invasive species through intentional manipulations of symbiotic relationships that result in either reduced competitiveness of invasive species or increased productivity and fitness of non-invasive plants (e.g., plants recruited after habitat restoration efforts). For example, if it is shown that fungal endophytes enhance the competitive capacities of an invasive plant species, encouraging the growth of antagonistic bacterial endophytes through exogenous applications may be explored as a way to truncate benefits stemming from fungal endophytes. This strategy is successfully being employed in crop plants to eliminate toxic endophytes (Bacon and Hinton, 1999).

Manipulation of the plant microbiome is a strategy that may be used to alter the competitive capacity of plants. Strategies to encourage or discourage specific microbes that impact plant performance may be employed, either to reduce competitiveness of the invader or to increase the resilience of native species. Such a microbiome manipulation strategy has been successfully explored with human health issues and serves to illustrate the promise of such an approach. Although many now recognize the importance of diet in directly manipulating the gut microbiome of humans and other animals (Muegge et al., 2011), other manipulation strategies with humans such as fecal transplants are gaining scientific credibility and public acceptance (van Nood et al., 2014). In fecal transplantation therapy, complex gut microbiomes from a healthy donor are introduced into the colons of patients suffering from intestinal infections. Often such probiotic manipulations reverse the trajectories of sick patients in a matter of days, restoring them to health (de Vrieze, 2013). Similar probiotic therapies involving plants have been used in agriculture for many years, whereby the introduction to soils of complex microbiomes from naturally disease suppressive soils (Chaparro et al., 2012) or from disease suppressive organic amendments (Hadar and Papadopoulou, 2012) have altered plant health trajectories by altering microbial species in the soil microbiome. These species are then recruited to the plant as endophytes and epiphytes. Similar plant and/or soil microbial manipulations could also be possible to alter invasion trajectories of introduced plant species.

We recognize that determining the role of the various microbial species in the success of invasive *Phragmites* or other plant species is complex, given the large number of biotic and abiotic variables involved. It is widely appreciated that beneficial symbionts such as rhizobia and mycorrhizae can enhance host nutrition, growth, and stress resistance, while pathogens have opposite effects. Beneficial plant-growth promoting bacteria, primarily found in the soil environment, are also known from many agricultural and natural systems where they help improve the growth and vigor of host plants (Compant et al., 2010). In agricultural systems, specific microbes are often used as bioinoculants to enhance crop productivity or to reduce pathogen and pest damage (Nelson, 2004; John et al., 2011). For example, plant growth-promoting rhizobacteria are applied directly to seed or to the soil when planting to ensure inoculation with the most beneficial strains (Kaymak, 2011). Plant-growth promoting fungi such as *Trichoderma* species can also have similar positive effects on plants distinct from other plant-symbiotic fungi such as mycorrhizal fungi and foliar fungal endophytes (Harman et al., 2004; Contreras-Cornejo et al., 2009). Colonization of roots by plant-growth promoting fungi can enhance resistance to pathogens and abiotic stresses, nutrient uptake and the productivity of crops. However, the biological roles of most host-associated microbes are unknown. Initial research to identify the most common and widespread microbial taxa found in the rhizosphere or within the target species can guide subsequent evaluation of microbial impact on host plants. Microbial taxa common among target plants growing within many populations throughout the landscape are more likely to influence landscape-scale competitiveness than taxa only observed in a limited number of plants. In the case of invasive plant species, the most prevalent and beneficial microbes could be targeted for control through chemical or biological treatments to reduce the growth and vigor of the invasive plant indirectly.

### Forming a science agenda

**Figure 2:**
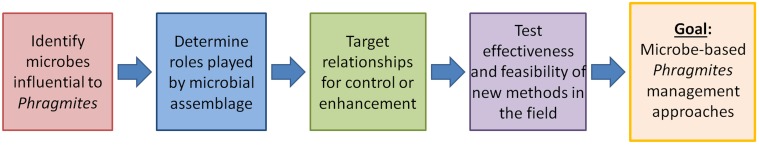
**Conceptual strategy for developing a microbial-based management approach to invasive plant species (e.g., *Phragmites australis*)**.

There has been some recent work highlighting the role of the microbial community in invasion success (highlighted above), but significant information gaps need to be filled before microbial-based control measures can be developed. To accelerate this development, we propose that a series of strategic actions can be used to ensure that the correct microbes are being targeted and that the desired results are achieved. Figure 2 provides a foundation of sequential steps to guide the development of microbial-based control strategies for invasive plant species. Similarly, it could be used as a guide to develop probiotics that promote the growth of native plant species.

1. ***Identify and characterize microbes influential to target invasive and non-invasive plants***—To design and implement an effective microbiome manipulation strategy, the microbial constituents relevant to the *invasive* plants of interest must be characterized. The host range, tissues colonized, mode of transmission (e.g., vertical, horizontal), assemblage diversity, temporal variability, and other criteria can help describe the local microbiome. A complete inventory will include documentation of any relevant rhizospere microbes, such as *Oomycetes* or plant growth promoting and endophytic *Bacillus* spp. (Gond et al., 2014), and whether significant interactions among endophytes exist. This step establishes the foundation on which the remaining steps are based.
  It is likely that *native* plant communities also are intrinsically linked to fungi and bacteria. Thus, it is also important to identify which endophytes are common in native species and initiate studies that will allow forecasting of possible behavior and outcomes from either a common species or an interaction of species during a specific growth phase. Results could guide targeting of specific life-stages, both pre-infection and post-infection, to maximize treatment response.
2. ***Determine roles played by the microbial community***—Once the target microbiomes are characterized, it is necessary to examine the benefits or other effects that they confer to the plants. Specifically, this step involves identifying the functional roles of identified microbes and exploring how they affect plant growth, development, and tolerance to extreme conditions (all characteristics that contribute to a plant's competitive ability). Similarly, examination of how identified microbes affect the function and competitiveness of native plants.
3. ***Target relationships for control or enhancement***—Once the microbial constituents and their roles are identified, the most influential relationships could be targeted for control or enhancement. Specifically, this stage will involve determining if endophytes can be controlled, how control treatments impact both target and non-target species, and how treatments alter competitive ability. Endophytes in target native plant species require a different approach focused on determining whether native species can be inoculated with beneficial endophytes (i.e., probiotics) and if inoculation will increase competitive abilities compared to invasive plants.
4. ***Test effectiveness and feasibility of new methods under field conditions***—After critical microbial assemblages are identified and targeted for control or enhancement, new management methods need to be developed and field-tested to characterize effectiveness, cost-efficiency, and risk through space and time. For example, tests of treatment specificity will characterize potential impacts of a control method on non-target organisms and environments. This step also involves examining the feasibility of scaling up to the landscape level and exploring the regulatory and financial aspects of new control (or enhancement) methods.

## A case study on the invasive common reed: creating a science agenda for managing invasive *Phragmites australis* through microbial intervention

### Ecology of *Phragmites*

**Figure 3:**
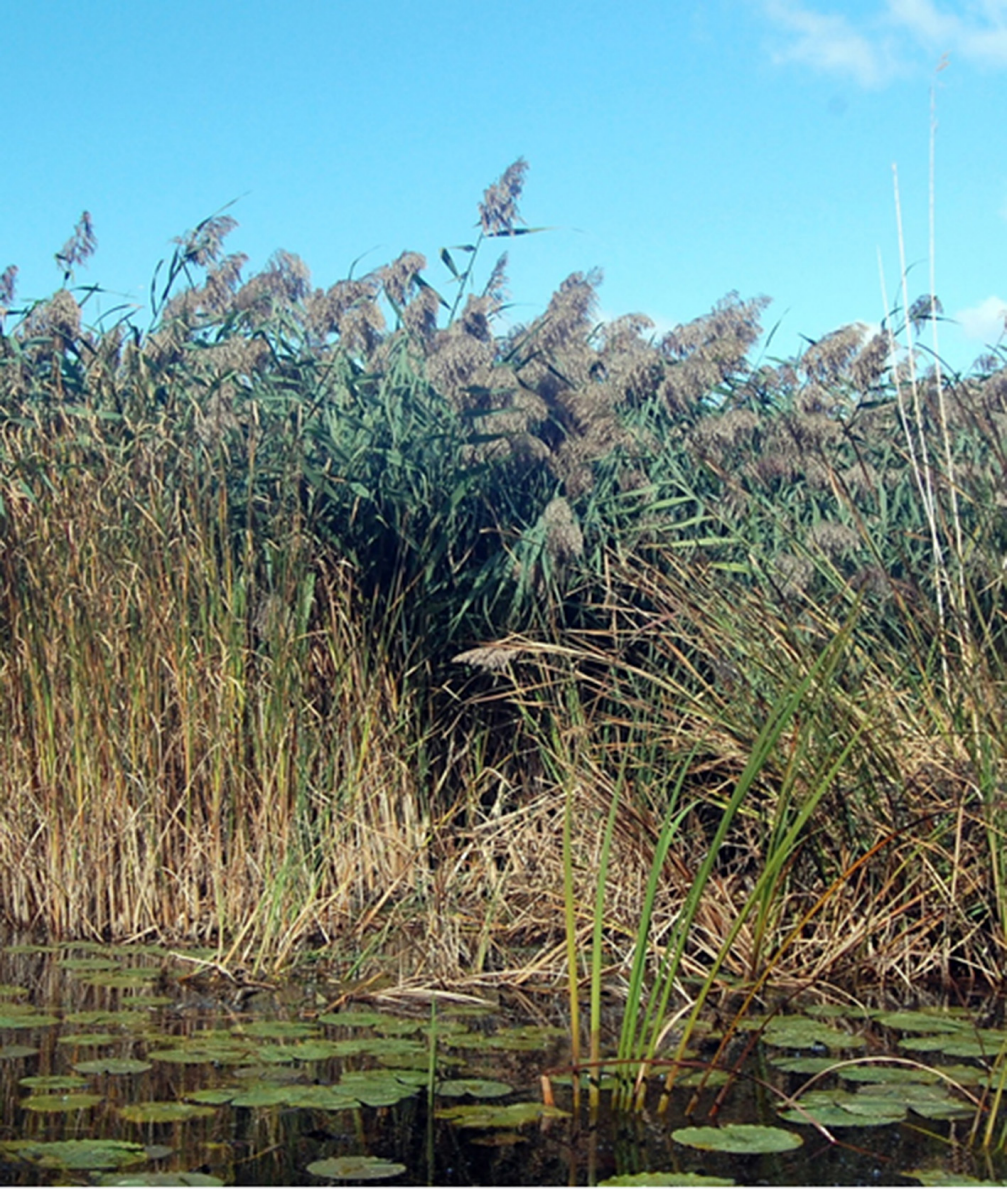
**Invasive *Phragmites australis* in a Great Lakes coastal wetland**.

The invasive form of common reed (*Phragmites australis*, hereafter referred to as *Phragmites*) is a tall non-native perennial grass often growing in dense clones throughout North American wetlands (Figure 3). Although a native subspecies of *Phragmites* (*Phragmites australis* spp. *americanus*; Saltonstall et al., 2004) has been present in North American wetlands for thousands of years, recent aggressive proliferation has been attributed to a non-native, invasive subspecies (*Phragmites australis* spp. *australis*), also known as haplotype M. The invasive *Phragmites* was introduced into North America from Europe near the beginning of the 1900s and has since been aggressively replacing the native type (Saltonstall, 2002; Mozdzer et al., 2013) and displacing native wetland plant assemblages. It is widely distributed and has been found in each state within the contiguous United States, is now established across the whole Great Lakes basin (Mal and Narine, 2004; Trebitz and Taylor, 2007; Tulbure et al., 2007; Bourgeau-Chavez et al., 2013), and can be found throughout southern Canada (Saltonstall, 2002).

This highly invasive plant spreads rapidly through seed dispersal, stolons, and rhizomes. *Phragmites* invasion displaces native plants and decreases wetland biodiversity, primarily because of its aggressive root system and tall, dense canopy that shades out other wetland plants (Chambers et al., 1999). It also may exude phenolic gallic acid as a form of allelopathy (Rudrappa et al., 2007; Bains et al., 2009), but the significance of that trait is not clear (see Weidenhamer et al., 2013). The presence of *Phragmites* is known to impair recreational use of wetlands and shorelines, decrease property values, increase fire risk, and reduce public safety when proximity to roads disrupts driver visibility (Warren et al., 2001; Mal and Narine, 2004; Trebitz and Taylor, 2007; Kettenring and Adams, 2011). A few studies describe some positive effects of *Phragmites*, including improved oxidation of the substrate and quality of the sediments (Tulbure et al., 2012), filtration of nutrients from agricultural lands (Kettenring et al., 2012), and providing beneficial habitat for the American bullfrog population (Rogalski and Skelly, 2012). However, invasive *Phragmites* is considered a significant ecological and economic threat by the public, NGOs, and governmental agencies (Meyerson et al., 2000; Great Lakes Restoration Initiative, 2014).

### Current management of *Phragmites*

Current management approaches can be effective in the short term, but there are currently no clear means to stop *Phragmites* invasions completely (Marks et al., 1994; Warren et al., 2001). Conventional *Phragmites* management typically involves the application of several strategies (chemical, mechanical (cutting and burning), and hydrologic) used in combination over a long period of time (Hazelton et al., 2014). This integrated approach is considered to be the most effective, yet when employed independently, these strategies may enhance *Phragmites* growth. Specific management protocols depend on many factors, including patch size and management agency capacity, but in general, repeated application of herbicides (glyphosate and imazapyr), followed by removal of biomass by burning or mowing is an effective *Phragmites* management approach (Carlson et al., 2009; Michigan Department of Natural Resources, 2010; Hazelton et al., 2014). While this protocol has been successful at reducing *Phragmites* in the short term, it is expensive, time-consuming, and generally not sustainable in the long term. Herbicides can also have negative impacts on the surrounding environment (Back and Holomuzki, 2008), and their application often draws negative social attention (Blossey, 1999). Furthermore, aerial and over-water application of herbicides is prohibited in Canada. Because current management methods are unsustainable and not available to all resource managers, new microbe-based strategies are being investigated.

### The microbiome of *Phragmites*

Baseline assessments of *Phragmites*-associated endophytes offer a foundation for exploration of potential control methods based on microbiome manipulations. Commonly, the freshwater and saltwater wetlands invaded by *Phragmites* harbor high levels of microbial diversity and activity (Gutknecht et al., 2006; Stephenson et al., 2013). As a result, diverse symbiotic interactions of *Phragmites* with eukaryotic and prokaryotic microbes are likely to occur. Although not always easily detectable (Lambert and Casagrande, 2006), ample evidence exists that *Phragmites* harbors rich endophytic fungal (Angelini et al., 2012; Fischer and Rodriguez, 2013) and bacterial (Li et al., 2013; Ma et al., 2013) communities comprised of both mutualists and potential pathogens. Equally significant are the epiphytic prokaryotic (bacterial and archaeal) communities (Llirós et al., 2013; Zhang et al., 2013) and fungal communities (Wirsel et al., 2001; Van Ryckegem and Verbeken, 2005). In addition, *Phragmites* is known to support oomycete communities (water molds, Wielgoss et al., 2009; Nelson and Karp, 2013). However, despite the detection of many known mutualistic and pathogenic symbionts associated with *Phragmites*, the specific roles of nearly all of these *Phragmites-associated* microbes have not been evaluated. The exceptions are a few *Phragmites*-associated rhizosphere bacteria (Reed et al., 2005) and fungi (Ernst et al., 2003) that have been shown to enhance plant growth.

A diversity of known pathogenic fungi (Ban et al., 1998; Mazurkiewicz-Zapalowicz, 2010) and oomycetes (Nechwatal et al., 2008; Nelson and Karp, 2013) have also been described in *Phragmites* populations found in both Europe and North America. Yet, despite the fairly extensive list of putative foliar- and root-infecting pathogens, virtually nothing is known about their virulence to either native or non-native *Phragmites* haplotypes, and little mechanistic understanding is known about how they might influence invasive success. Therefore, screening for well-studied microbes, like *Bacillus* spp. that are known to promote growth and resistance to biotic and abiotic stresses in a range of plants (Gond et al., 2014; White et al., 2014) could provide initial insight into the relationship between *Phragmites* and pathogenic microbes. These microbes are better understood in terms of their mechanisms of activity in plants and therefore could provide important targets for altering the competitiveness of invasive *Phragmites*.

Detection and examination of mutualistic or pathogenic endophytes in *Phragmites* is complicated by the fact that plant-growth characteristics associated with host variation may be completely distinct from those influenced by environmental or physiological adaptations (Lissner et al., 1999; Meyerson et al., 2000; Saltonstall et al., 2004). Since microbiome associations are very genotype specific and there is a suite of *Phragmites* lineages in North America, host variation must be addressed in any investigation endophytic and epiphytic microbes and their effect on the invasive success of *Phragmites*.

### Microbial-derived benefits to *Phragmites*

Microbial interactions are thought to convey benefits to invasive *Phragmites* through enhanced nutrient processing capabilities and increased tolerance to environmental and habitat disturbances. *Phragmites* is well-adapted for growth in nutrient-rich habitats but is somewhat plastic in that it grows at low nutrient levels also (Mozdzer and Megonigal, 2012). Although *Phragmites* commonly can be found in low nutrient soils, it grows best at fertile sites (Romero et al., 1999). The capacity of *Phragmites* to cross a range of soil nitrogen concentrations could be related to maintenance of microbial functional diversity with respect to nitrogen processing in multiple parts of the nitrogen cycle (Li et al., 2013). A species of the fungus *Stagonospora*, for example, was found to be a common growth-promoting endophyte of *Phragmites* (Ernst et al., 2003), so it is possible that this fungus or other fungal species could effectively replace the nutrient absorption function of AMF. Because AMF have obligate associations with plants, the limited support provided to these fungi by *Phragmites* may also provide a possible explanation for the slow re-colonization by native plants in managed marshes that were previously dominated by *Phragmites* and have depleted levels of AMF in the soil (e.g., Tanner and Gange, 2013). Holdredge et al. (2010) found that native *Phragmites* was much more heavily colonized by AMF, suggesting that it would benefit more from increased abundance of AMF than would the invasive strain.

*Phragmites* is remarkably tolerant of and resilient to a variety of environmental and habitat disturbances (Hellings and Gallagher, 1992; Minchinton and Bertness, 2003; Silliman and Bertness, 2004; Li et al., 2010), but little is known about how endophytes may mediate such responses. Chen et al. (2012) surveyed endophytic bacteria associated with *Phragmites* and evaluated their capacities to degrade pesticides and other pollutants. They proposed that endophytic bacteria could enhance the capacity of *Phragmites* to detoxify polluted waters. The presence of such bacteria may also contribute to the tolerance of *Phragmites* to grow in contaminated sites, where this may contribute to its invasiveness (Meyerson et al., 2000), although endophytic microbial population shifts are observed along with environmental changes (Ravit et al., 2007; Ma et al., 2013). Overall, the presence of endophytes leads to an increase in plant-produced antioxidants and general up-regulation of other stress-defensive mechanisms that may enhance stress tolerance and increase invasive success (Waller et al., 2005; Hamilton et al., 2012; Torres et al., 2012).

### *Phragmites* management via microbiome manipulation

The control of *Phragmites* in North America has become very resource-intensive and difficult to maintain. A recent study of land managers found that, between 2005 and 2009, about $4.6 million was spent annually on mechanical and chemical control of *Phragmites* on over 80,000 hectares in the United States, but there is no significant relationship between the resources invested and control success (Martin and Blossey, 2013). These findings indicate that there is a need for improved control methods using more effective and sustainable approaches.

Successful management of invasive *Phragmites*, like other invasive plant species, would benefit from an integrated management approach that engages multiple stakeholders and can attract substantial long-term funding. As a result of this need, researchers at the U.S. Geological Survey partnered with the Great Lakes Commission to use principles of the collective impact approach (Kania and Kramer, 2011) to establish the Collaborative for Microbial Symbiosis and *Phragmites* Management (hereafter called the *Phragmites* Symbiosis Collaborative or PSC). The PSC was initiated in February 2013 to advance microbe-based research on the control of invasive *Phragmites*. This powerful collaborative approach is new because it brings together an international group of researchers from many disciplines and agencies to focus on the development of an innovative microbe-based control strategy for invasive *Phragmites*.

The objectives of the PSC are to establish the current state of the science, identify research gaps, and develop a science strategy (i.e., research agenda) to guide and support research on microbial symbiosis to maximize collective progress toward an integrated *Phragmites* control and habitat restoration strategy. The PSC agenda (Table 1) includes explicit steps that guide the scientific community in the development of new control methods based on microbiome manipulation. These mutually reinforcing steps target the competitive abilities of invasive *Phragmites*, as well as lay out principles and approaches that will serve as a foundation for application of microbiome manipulations to other invasive species. Using the five conditions of collective impact (a common agenda, a shared measurement system, mutually reinforcing activities, continuous communication, and a backbone support organization) to plan and support the initiative (Kania and Kramer, 2011), this collaboration of scientists is fostering progress toward a broader overall vision to maximize the collective impact of individual research efforts.

**Table 1:** **Specific tasks outlined by members of PSC to guide research to support *Phragmites* management using microbial symbiosis**.

**Science agenda**	**Tasks**
Microbial inventory	(a) Gather data on the composition and transmission method of epiphytic and endophytic microbes associated with *Phragmites* populations
	(b) Determine the variation of the *Phragmites* microbiome in time and space (e.g., within a stand, by site) or time (e.g., over plant life cycle, age of *Phragmites* stand)
	(c) Explore the relevant pathogenic microbes in *Phragmites* communities and interactions that may exist with mutualistic microbes
	(d) Characterize the microbiomes of target native plant species to determine if there is a common core group of taxa from which to explore their significance in a probiotic management approach
	(e) Determine variation in native species microbiomes in space, by species, or by growth stage to allow some predictive patterns that may inform the timing of a manipulative strategy
	(f) Compare the endophytic communities of invasive *Phragmites* to that of native *Phragmites*
Benefits of microbes	(a) Test the plant response of *Phragmites* when inoculated by particular microbe or set of microbes
	(b) Determine endophytes that impact growth rate, biomass production, tolerance to stress, or other characteristics that may provide a competitive advantage
	(c) Assess the impacts of inoculants on *Phragmites'* competitive abilities
	(d) Determine the impact of *Phragmites*-associated pathogens on native plant communities
	(e) Identify particular microbes associated with *Phragmites* or with native plants that increase the relative competitiveness of native wetland species in the presence of *Phragmites*
	(f) Identify individual microbes or microbial consortia that impact plant developmental pathways (e.g., nitrogen-fixing bacteria)
Targeting relationships for control	(a) Test microbial sensitivities to inhibitors (e.g., fungicides or antibiotics)
	(b) Determine the selectivity of microbial inhibitors for particular groups microbes
	(c) Test endophyte sensitivity to treatments with limited environmental impact
	(d) Determine the competitive outcomes of *Phragmites* with native plants following the elimination or suppression of selected microbes
	(e) Determine competitive outcomes of *Phragmites* with native plants with the inoculation of mutualistic microbes or with the elimination or suppression of pathogens (f) Explore mechanisms that underly reductions in *Phragmites* competitiveness
Test control methods	(a) Analyze considerations for scaling up to landscape-level application of microbial-based control methods
	(b) Perform analysis for appropriate regulatory bodies and involve regulators in discussions and planning
	(c) Determine impacts of microbial manipulations on non-target species
	(d) Determine the direct environmental impacts of the method of manipulation (e.g., fungicide, boric acid)
	(e) Assess costs associated with microbiome manipulation management strategies
	(f) Explore optimal management efficacy at short- and long-term time scales

## Summary

Microbial communities affect plant health and productivity in many ways and likely contribute to the competitive success of invasive plant species. The symbiotic relationships between invasive plant species and their associated microbes offer a new target for development of control methods and management strategies. However, the spatial and temporal composition of microbial communities in invasive plants, as well as the roles they play in plant competition, are not well-characterized. Similarly, approaches for microbiome manipulation as a form of invasive species control are under development. Therefore, this paper reviewed the relevant science relating to plant-microbial interactions and identified a conceptual strategy for uncovering the microbial interactions that could influence invasion success. A case study on the invasive grass *Phragmites australis* showed how the collective impact approach can be applied to create a science agenda for development of microbe-based control strategies. The steps outlined in this case study will serve as both a foundation for similar microbe-based control efforts targeting other invasive species and a model of the collective impact approach that can be applied to other avenues of research and management.

## Author contributions

The manuscript was drafted by KK, CB, WB, HB, KC, ML, MM, EN, MT, and JW with editorial remarks from DW.

### Conflict of interest statement

The authors declare that the research was conducted in the absence of any commercial or financial relationships that could be construed as a potential conflict of interest.

## Glossary

***Arbuscular mycorrhizal fungi (AMF)***: Group of fungi in order Glomales that colonize plant roots and enhance plant growth by increasing absorption of minerals from soils.
***Biotic resistance hypothesis***: States that species-rich communities are more resistant to invasion because they are able to use the resources more efficiently than communities with low species richness.
***Biotrophic***: Describes an organism that can live and multiply only on another living organism, such as parasitic or symbiotic bacteria and fungi.
***Class 1 Fungal endophytes***: Fungi in the family Clavicipitaceae that have a narrow host range (grasses) and colonize shoot and rhizome tissues.
***Class 2 Fungal endophytes***: Non-clavicipitaceous fungi that have a broad host range and colonize shoot, root, and rhizome tissues.
***Class 3 Fungal endophytes***: Non-clavicipitaceous fungi that have a broad host range and colonize above-ground tissues.
***Class 4 Fungal endophytes***: Non-clavicipitaceous fungi that have a broad host range and colonize root tissues.
***Collective impact***: The commitment of a group of actors from different sectors to a common agenda for solving a complex problem; individual impacts are multiplied through collective effort.
***Ecological resistance***: Reduced invasion success in a native community associated with multiple biotic processes, including predation, competition, herbivory, or disease.
***Endophyte***: An organism, often a bacterium or fungus, that lives within the tissues of living plants; relationships with plant vary from symbiotic to nearly pathogenic.
*Enemy release hypothesis:*: States that the success of exotic organisms is due to escape from natural enemies within their native range.
***Epichloë***: A genus of systemic and constitutive fungal symbionts of cool-season grasses.
***Epiphytes***: Microbes that grow and persist on plant surfaces.
***Haplotype***: A designation based on a group of genes within an organism that was inherited together from a single parent.
***Horizontal transmission***: Transmission of an infective agent (e.g., microbe) between individuals in a population.
***Microbiome***: All of the microorganisms that associate with another organism either externally or internally.
***Mutualism***: A relationship between two organisms in which both benefit from the association.
***Mycorrhizal***: Refers to fungi that associate with plant roots and facilitate the uptake of nutrients.
***Oomycete***: Eukaryotic microorganisms within the kingdom Chromista, characterized by biflagellate swimming zoospores and the formation of oospores.
***Pathogen***: A microbe capable of causing host damage.
***Phyllosphere***: Surface of plant leaves that may be colonized by microorganisms.
***Phytosphere***: Plant ecosystem including the exterior and interior of both aboveground and belowground portions of plants.
***PSC***: Collaborative for Microbial Symbiosis and *Phragmites* Management.
***Rhizosphere***: Area of soil surrounding plant roots where the abundance and activity of microorganisms is elevated due to root carbon deposition.
***Symbiosis***: Interaction between two different organisms living in close association, typically to the advantage of both (includes mutualism, commensalism, parasitism).
